# American Proctologic Society

**Published:** 1901-07

**Authors:** 


### SOCIETY REPORTS.

### AMERICAN PROCTOLOGIC SOCIETY.

*Third Annual Meeting, St. Paul, Minn., June 4, 1901.*

The meeting was called to order by the President, Dr. James P. Tuttle, New York. After the reading of the minutes of the previous meeting and receiving the Treasurer's report the society entered upon scientific business, abstract of which appears below.

At the conclusion of the scientific business the following officers were elected to serve during the coming year:

President—Dr. Thomas Charles Martin, Cleveland.

Vice-President—Dr. George J. Cook, Indianapolis.

Secretary and Treasurer—Dr. William M. Beach, Pittsburg.

Executive Council—Dr. J. M. Mathews, Louisville, Ky.; Dr. James P. Tuttle, New York; Dr. J. Rawson Penington, Chicago.

Prof. Dr. Sonnenberg, Berlin, was elected to honorary membership in the society on motion of Dr. William M. Beach, Pittsburg.

The society adjourned to meet at Saratoga, New York, in June, 1902.

Dr. James P. Tuttle, New York, N. Y., read a paper on

### MALIGNANT TUMORS OF THE RECTUM.

In his consideration of this subject, the essayist divided them into four classes—connective, epithelial, muscular and irregular tissue growths.

It was stated that with those in his own practice, together with those mentioned in the literature of the subject, there were twenty-nine of the melanotic type and fourteen of the non-melanotic.

Sarcomas occur in the rectum as irregular deposits beneath the mucous membrane, in shape being round, elliptical, and sometimes resembling a hypertrophied tonsil. They rarely, if ever, assume the smooth, plaque-like form of deposit, such as is seen in carcinoma. The surface being always rough, unequal, "muriform," and the mucous membrane movable over the growth in its earlier stages, is a condition which distinguishes them from carcinoma.

They originate in the sub-mucosa, and as they grow may appear as sessile tumors, and eventually develop a distinctly polypoid shape. They may also appear as a general fibrous thickening of the wall, and be mistaken for a simple inflammatory stricture.

The mucous membrane covering sarcomas is comparatively normal, although if the tumor becomes very large the membrane may become congested, edematous, or ulcerated, and even adherent to the growth through inflammatory processes. Sarcomas in the rectum may occur single or multiple, and vary in size from that of a hazelnut to a good-sized orange. One case reported was as large as a cocoanut.

Sarcomas of the rectum present a variety of colors, generally that of the normal mucous membrane, although sometimes they are dark red, grayish-black, bright red, pale yellowish-pink, or as black gangrenous masses. Often in the multiple form, the different tumors will present varying appearance.

Sarcomas may occur at any portion of the rectum or sigmoid, but the majority are situated low down near the anal margin.

Sarcomas differ from carcinomas by their rapid growth. Differing from sarcomas in other parts of the body, these sarcomas are said to have a distinct tendency toward ganglionic infection.

Metastasis is one of the chief characteristics of sarcomas of the rectum. If the growth is primary, all possibility of metastatic deposit should be eliminated, or else the operation will be of no avail.

A complete *résumé* of the histology was given under the following heads: round or globe-celled sarcomas, spindle or fusiform, giant cell, alveolar, and mixed.

Melanosis does not alter the type of the tumor or change the character of its component parts. It takes place in all types, and may involve but one part of a tumor, or only one or two tumors where they are present in multiple form.

Sarcomas of the intestine always develop from sub-mucosa, and ordinarily do not affect the mucous membrane until, by pressure, tension, and ulceration, through friction and infection by the fecal mass, it may become involved. The causes and influences which bring about the production of sarcoma are as little known as those of carcinoma.

Age cannot be proved to have any direct influence, although it occurs more often late in life, and there is apparently no relationship between the sexes and this disease.

Symptoms are at first very vague. There may be a sense of fullness, or a feeling of the presence of a foreign body, or the first symptom may be bleeding and discharge of mucus.

The protrusion of sarcomatous tumors is more frequent than that of carcinoma, but less so than in other forms of rectal neoplasms.

There is no odor peculiar to sarcoma. After ulceration has occurred and there is a production of pus, the odor changes to that of decomposing tissue, but never assumes that peculiar characteristic and disgusting odor which is found in carcinoma of the rectum.

If the sarcoma is low down and involves the sphincter, producing traction and pressure, the patient may suffer considerable pain. But if it is high up and of an infiltrating form the patient may go to the very door of death without any knowledge of its existence.

The state of the bowels in sarcoma of the rectum varies according to the type of the tumor. There may be either constipation or diarrhea. The latter may be caused by the mobility of the growth and its location near the margin of the anus. Constipation may be caused upon mechanical grounds.

Flatulence, indigestion and loss of appetite are associated with sarcoma of the rectum as they are with all other neoplasms of this organ.

Cachexia is not well marked. Reflex digestive disturbances are noted. Decrease in strength, loss of flesh, swelling of the feet and abdomen rapidly succeed one another, when the sarcoma is once well developed.

Dysuria is frequently present. The lungs and pleura may become affected.

The diagnosis of this condition lies between carcinoma and villous tumor. It is less sessile than carcinoma, and less pedunculated than adenoma. It is more firm than adenoma and has a less degree of induration than carcinoma.

In its attachment its roots do not spread out, producing that general infiltration of the mucous membrane that one finds in carcinoma. Its attachment is very abrupt. To the touch it is more undulating and irregular than carcinoma, but has not the granular and dendritic divisions which one finds in villous tumor and in adenoma. The latter occur largely in children, whereas sarcoma is a disease of middle or advanced life.

When it is a question between sarcoma and multiple adenoma, the multiplicity of the growths, the excessive diarrhea, together with the comparatively fair condition of the patient's health, may be mentioned upon the side of adenoma.

Between sarcoma and carcinoma, the distinct odor of the latter is enough to make the decision positive.

In the early stages of the disease the fact that the mucous membrane moves easily over the growth distinguishes it almost positively from carcinoma.

The final test depends upon the microscopic examination of a section from the real substance of the tumor itself.

Personally the reader was opposed to making an incision to obtain the section unless the case was an operable one and the patient consented to an operation if the microscopic examination showed the necessity for one.

The treatment of the disease consists in the radical removal of the growth. A ligature to pedunculated sarcomas ought never to be considered. If the growth is single and in the wall of the rectum a posterior proctotomy may be done. If it is diffuse, involving the entire circumference of the rectum, total excision of the organ is the only recourse.

While there is some evidence of the value of the serum-therapy in the treatment of the sarcomas elsewhere, the advocates of this method lend no encouragement by their results in the treatment of this condition in the rectum.

Artificial ani may give great relief in carcinoma, but it neither relieves nor checks the progress of sarcoma.

### PRESIDENT'S ADDRESS.

The President, Dr. James Tuttle, New York, in his annual address, discussed the various phases as to whether or not it would be advisable for the American Proctologic Society to continue as an independent association or apply to the American Medical Association for admission as a proctologic section. He spoke at length of the advantages of meeting at the same time and place of the assembling of the great medical body. He spoke of the desirability of being brought into closer contact with the general profession, from whom they had much to learn, and to whom many debts to pay.

The profession should be educated to realize the fact that there is more in proctology than they now believe.

The average practitioner's conception of this subject is that it consists in tying off piles, cutting through fistulas, and stretching the sphincter muscles for fissure.

Year after year the speaker stated that men attended his clinics who said that they were determined to make a specialty of rectal diseases. They expected to become accomplished specialists in from three to six weeks. They wanted to see as many operations for piles as possible during that time. They didn't mind if a fistula was thrown in for good measure, but "piles" was their conception of proctology. The most carefully prepared lecture or demonstrations of new methods of diagnosis and the teaching of intestinal pathology are all lost upon them, for they are there to learn how to treat *rectal* diseases—*i. e., piles.*

When they have spent three or four weeks in this deep and profound study these men go back home full-fledged rectal specialists, and sometimes are made professors of the branch in some provincial college.

The speaker did not want for one moment to reflect upon those noble practitioners of general medicine who attend postgraduate schools intent upon learning how to diagnose and treat disease. All honor is given to these men who know their deficiencies, who sacrifice so much to keep abreast with the progress in medicine, and who go back to their homes and unpretentiously give their patients the benefits of the knowledge gained by honest study.

The essayist scored the mushroom specialist and the advertising charlatan, who, he said, were molding public opinion upon proctology. They publish their advertisements and scatter their pamphlets everywhere, until the public commence to make their own diagnosis. The family doctor was said to be partially to blame for this condition of affairs, as he so often diagnoses these conditions without examination.

The advertising charlatan would have the public believe that the regular physicians never make a study of rectal disease, that his instruments are patented, and that successful methods of treatment are known only to him.

The reader stated that it was the aim of the American Proctologic Society to show to the medical profession, and through it to the public, that there is something more in the subject, and questioned if there was a better way of accomplishing this than by interesting the A. M. A. sufficiently in it to establish a proctologic section, where they could meet the general practitioner, tell him what they are doing, and learn from him his needs.

The reader favored the attempt to organize a proctologic section.

Another point considered in the address were the qualifications for membership in the society. In closing the speaker said:

"All over the country there are springing up specialists in rectal diseases, made by short terms of study at some post-graduate school or by being elected professors of this branch in some small college. As a rule, they are without experience or learning in the branch, and accept the position, simply on account of the title and emoluments. On the other hand, there are a large number of general surgeons whose hospital appointments require their doing large amounts of rectal surgery. The first class will be knocking at your doors for admission, but they bring no offerings in the fruits of their labors. The latter class will only come by invitation, but when they do, they will bring a rich experience and many practical observations gained in general surgery, but useful to the specialist. Holding a chair in some little medical college does not entitle a man to membership in this society, and being a general surgeon or practitioner should not debar him. Let us select our members with such care that in the future we can never wish that this or that one had not been let in."

It was moved that the President's address should be open for discussion.

On motion of Dr. Martin, the Chairman was authorized to appoint a committee to consider the President's address, and select a time for its discussion. At a subsequent session the committee reported the following resolution:

"Your committee would recommend that a vote of thanks be tendered Dr. Tuttle as a recognition of his valuable contribution to the literature of malignant disease of the rectum.

"Your committee are agreed that this time is inopportune for negotiation for admission to the American Medical Congress, and that at present it is inadvisable to attempt the organization of a section on proctology in the American Medical Association; therefore, we recommend the adoption of the President's suggestion, that our next meeting be at the time and place of the next meeting of the A. M. A.

JOS. M. MATTHEWS,
THOS. CHAS. MARTIN,
Committee."

When the subject was opened for discussion President Tuttle moved the adoption of the resolution, thus reversing the opinion expressed in his paper. He had come to the conclusion that the interests of the specialty could be best subserved by remaining an independent society. Dr. J. M. Mathews, of Louisville, Ky., also took this stand. After some further discussion the resolution was adopted as read.

DISEASE OF THE SIGMOID, by Dr. George B. Evans, Dayton, Ohio, was then read by the author.

The essayist discussed the question as to whether or not the rectum was the receptacle for feces, or whether the latter is arrested, detained, and accumulated in the sigmoid flexure of the colon. The reader inclined to believe that the rectum was the receptacle.

From its situation and anatomical relations, the reader was convinced that the sigmoid is oftener the seat of obscure abdominal diseases than has generally been suspected. In appendicitis there is often reflected pain over the whole abdomen, often in the left iliac region over the sigmoid. Now if this is true, the converse is also true. This point was illustrated by reference to a patient who had a distinct history of appendicitis. The condition was promptly relieved by flushing the sigmoid and colon with large quantities of hot boracic solution. This treatment was advised in all cases of supposed appendicitis. It can do no harm and might do good.

Patients should be examined (1) by palpation and percussion, both in recumbent, with thighs well flexed, and in erect posture; (2) digital; (3) by a combination of the two; (4) by ocular examination, using the tubular speculum; (5) if the speculum should fail to enter the sigmoid then use the Wales bougie.

The reader was confident that many of the so-called catarrhal conditions of the bowels are congestions, if not inflammation, of the sigmoid, attended with large discharges of mucus, accompanied with pain over the abdomen. In these cases the sigmoid and colon are washed with hot boracic solution, using as much as one or two gallons. If the results are not satisfactory, three ounces of a 50 per cent. solution of fluid hydrastis are added and applied through a Wales bougie.

After the inflammatory changes have taken place the task is more difficult. If we find the stools mixed with pus and mucus there is ulceration, and if the rectum is healthy the trouble will be found in the sigmoid.

A case was reported illustrating this point which yielded readily to the flushing method.

Moderate exercise and a liberal and nutritious diet of milk, soft-boiled or poached eggs, and plenty of fresh air and sun baths are valuable adjuncts.

Syphilitic ulcerations should be treated constitutionally and locally as described. The inunction method alternately with the iodides, in conjunction with the passage of the bougie, is indicated. If the case be an operative one it is a question whether there should be a total resection, with end-to-end anastomosis, or a resection and then an anastomosis by passing the distal end of the sigmoid through a slit in the rectum, holding the sigmoid *in situ* by means of traction sutures passed through the muscular walls of the sigmoid, leaving the sutures long enough to emerge from the anus clamped by long artery forceps across it. If neither of these methods be practical, resort must be had to colostomy. The latter is palliative; the former radical, and the question is, who will advise it; when and under what conditions would it be justifiable?

The reader was also fully convinced that grippe was an important factor in sigmoiditis.

RECTO-COLITIS.

A paper with this title was then read by Dr. William M. Beach, Pittsburg, Pa.

The essayist described recto-colitis as a condition of the rectum and colon that generates functional derangements consequent upon varying degrees of inflammation of its mucous membrane.

A clear exposition of the anatomic elements of the gut and its auxiliary structures was given.

Omitting malignant diseases, recto-colitis was considered under the following stages: Congestion, atrophic catarrh, hypertrophic catarrh and ulceration.

Simple congestion due to engorged blood-vessels may be ephemeral and express itself locally in the form of dysentery and tenesmus and the excretion of an enormous quantity of mucus. This condition, if allowed to continue, becomes chronic, developing usually the hypertrophic catarrh; epithelium is shed, valves swollen and thickened, narrowing of rectal straits, diarrhea alternating with constipation.

Atrophic catarrh is usually accompanied by the constipated habit; dry hard stools, and minute anal fissures. Secretions are insufficient on account of gland impairment. Atrophic recto-colitis is rare.

The ulcer is the culmination of the inflammatory process, and in the experience of the reader rarely occurs above the sigmoid flexure.

The symptoms of recto-colitis are (1) local or physical; (2) constitutional or rational. The reader described them fully.

From the standpoint of the proctologist the question was asked, "Is chronic recto-colitis curable?" To answer this question it became necessary to study clinical experience. The writer called attention to cures reported by Matthews, Martin, and other proctologists, and referred particularly to one type, congenital or functional narrowing of the recto-sigmoid strait. In many of these cases the spasm is so strong when the instrument touches the inflamed surface that the strait is entirely closed; in others there is a prolapse of the sigmoid. A case was reported which had been referred to the reader by a neurologist. Examination revealed piles, and proctoscopy a very sensitive rectum covered with glairy mucus; the sigmoid strait narrow and spasmodic, with great pain at the upper part of the sacrum. The treatment consisted of a nightly administration of a saline; a daily injection of water, then, through the proctoscope, a mopping of the entire surface with sweet oil, and then through a narrow tube attached to a Davidson syringe a half pint is thrown into the colon, while the patient is in the Martin position. After peristalsis and tenderness subside, mildly astringent solutions are sprayed and rectal massage given. The reader had had most excellent results with the protargol spray.

Recto-colitis, due to mechanical obstruction or irritation, can only be relieved by removal of cause, to clear the field so that the remedies applied locally might be efficacious.

Abrasions, pin-point denudations of epithelium, should be touched with pure carbolic acid or solution of silver nitrate. If the mucosa presents a deeply injected appearance, bland remedies were thought to be more efficacious, such as albolence. Valvular hypertrophies were reduced by the use of a two-bladed divulsor, wrapped with cotton.

Internal medication was necessary to correct intestinal secretions and allay neurosal symptoms.

Recto-colitis, due to polypus, hemorrhoids, fistula or stiff valves, is to be cured by the removal of these conditions. In conclusion, the essayist said:

"Our discussion of recto-colitis consists in:

"1. That it is a condition of the rectum and colon of varying degrees of inflammation.

"2. A knowledge of the anatomical bearings of the rectum and colon is necessary to understand the symptoms and reflexes.

"3. The symptoms are local and systemic.

"4. Recto-colitis may be catarrhal or ulcerative.

"5. It may be acute or chronic.

"6. When dependent upon polypus, hemorrhoids, fistula, etc., the cure depends upon their removal.

"7. Chronic recto-colitis due to altered secretions, anemia and congenital narrowing of the sigmoid strait, is difficult to cure."

### ANAL POCKETS.

This paper was read by Dr. Louis J. Krouse, Cincinnati. The doctor first entered into a very exhaustive study of the so-called anal pockets, in which he gave the results of his own observations and those of other investigators. He discovered, by his researches, that these pockets were present in the rectums of the living to the extent of 80 per cent., but that they were entirely absent in the dead. In conclusion he said:

"That the so-called anal pockets may be the cause of certain diseases located in the lower outlet of the bowel, I cannot gainsay; but I believe that they are most likely the frequent predisposing cause of an irritable ulcer of the anus. If we examine the rectum in the quiescent state, when the bowels are empty, we find that the anus is closed; the anal valve and its corresponding sac are absent. But when the bowels move, the anal canal is opened and the anal valve becomes prominent, the same as would occur had an anal speculum been introduced and opened. Should a hardened fecal mass pass through the anal outlet, with a prominent pseudo-valve protruding, then this valve would most likely be caught by the moving mass and possibly be torn, producing what might be termed an irritable ulcer of the anus."

### THE TREATMENT OF RECTAL PROLAPSE.

Dr. J. Rawson Pennington, Chicago, read a paper on this subject.

In considering the treatment of rectal prolapse it is essential, first, to recognize the pathologic condition. The object of treatment is: (1) Reposition of the prolapse; (2) its fixation in the normal position; (3) prevention of recurrence.

He believes that some of the most important factors in the production of rectal prolapse are to be found within the intestinal canal, and considers the *plica transversalis recti et sigmoidæ* as one of the most, if not the most, important causative factors. He continued by saying that various procedures have been devised for the treatment of this malady, but to be successful the operation selected must be determined by the variety and specific conditions of the prolapse, otherwise it will be a failure.

Of these procedures he mentioned (1) those having for their object the production of adhesive inflammation between the coats of the intestinal walls; (2) narrowing the anal canal; (3) amputation; (4) reposition and bony fixation; (5) reposition and intra-abdominal fixation (colopexy or sigmoidopexy); (6) Thur Brantz massage; (7) electricity; (8) ligature. He recommended:

1. For prolapse of the mucous membrane only, reclining posture, adhesive straps, cauterization or amputation.

2. For reponable, non-ulcerated prolapse of all the coats of the rectum and colon invagination remove the cause, if possible, and try massage and electricity. Should these fail, then resort to colopexy.

3. For incarcerated irreponable ulcerated prolapse, circular resection, according to the technique of Mikulicz and Nicoladoni. The operation of colopexotomy, procto-coccypexy, procto-sacrococcypexy, procto-sacropexy, Gersuny's twist and the circular suture of Thiersch are rarely indicated.

A NEW METHOD FOR THE REMOVAL OF HEMORRHOIDS UNDER LOCAL ANESTHESIA

was explained by Dr. Thomas Charles Martin, Cleveland, Ohio.

He stated that non-malignant anal growths could be removed painlessly without resort to general anesthesia, by means of a technique which he would describe, provided it be performed by the trained hands of an operator who thoroughly understands the principles of infiltration anesthesia, and who, furthermore, has been sufficently persevering to master the difficulties encountered in the application of those principles to this operation.

Dr. Martin presented an instrument consisting of a hollow cone three and one quarter inches in length, three-quarters of an inch in diameter at its distal extremity, and one and three quarter inches in diameter at its proximal end. One quadrant of the cone is fenestrated. This is occupied by a movable blade with a serrated edge which makes contact with the cone's serrated edge. The movable blade is sheathed in the cone when the jaws of the clamp are separated. When it is introduced it may be made to receive the pile without irregularly expanding the anus. The great essential to painless manipulation of the sphincter is the even distribution of pressure throughout its circumference.

The patient should be placed in the Sims posture and the light focused on the field of operation.

The different tumors being located, the summit of each should be infiltrated with a one tenth of one per cent. solution of eucaine.

A very fine needle should be employed. Care should be taken, or else, instead of effecting an infiltration of the structure, the anesthetic may be driven at once into a blood space and directly into the circulation.

Each pile to be operated upon is seized by a curved hemostat, which should be surrendered to an assistant, who should radiate it from the anus and well out of the way of the operator.

The well-lubricated clamp should now be introduced into the anus with its blade pressing against the tumor which is first to be removed. When the instrument is buried to its shoulder the fenestrum should be opened, into which the hemorrhoid is pulled. The pressure incident to the introduction of the clamp is often sufficient to express the eucaine from the tumor, so that re-anesthetization becomes necessary in order to perform the manipulation necessary to carry the tumor completely within the clamp. The clamp should now be closed and locked and the growth cut away by means of scissors.

If secondary hemorrhage is feared, the wound should be lock-stitched with catgut. If it be of the connective tissue or fibrous variety, the pedicle should be cauterized. The wound should be treated as in any other surgical procedure. The use of this clamp gives the operator a clean field and a clear view. The pile is "dry docked."

This clamp demands that the wound shall be linear in form and parallel with the axis of the anus.

This method of clamp operation is inapplicable to inflamed or thrombotic piles.

Local anesthesia is a surgical refinement; skill in effecting it may be acquired only by the exercise of patience and practice.

Dr. George J. Cook, Indianapolis, discussed in a general way the employment of caustic agents in the treatment of hemorrhoids. His discussion was very thorough. The conclusion that he drew was that such agents for the most part should not be used, and that he recommended operative procedures whenever possible.

### FOREIGN BODIES IN THE RECTUM, WITH REPORT OF A CASE.

This paper, by Dr. Lewis H. Adler, Jr., Philadelphia, was read by title, but the case was deemed of sufficient interest to bring before the Society, even in the absence of the author.

A man sixty years of age was admitted to the Polytechnic Hospital, Philadelphia, December 1, 1900, with a history that he had been wearing for a long time an instrument which he called a pile-supporter, and that it had suddenly slipped within the bowel and could not be removed. Several attempts were made to remove the foreign body before succeeding. It was the handle and valve of a steam radiator pipe. The patient left the hospital the third day. Upon admission to the hospital the man's son informed the resident physician that his father was addicted to masturbation, and that he employed the wooden knob to further that habit. Subsequently the physician who had referred him to the hospital presented me with a large bent piece of iron rod, about fourteen inches long and one-fourth of an inch in diameter, which the patient had used for the insertion and withdrawal of the knob. The hooked iron rod and knob were exhibited to the Society by Secretary Beach.

